# “It is very convenient when it works – successes and challenges with welfare technology” - a qualitative study

**DOI:** 10.1371/journal.pdig.0000844

**Published:** 2025-04-24

**Authors:** Samantha A. Svärdh, Giana Carli Lorenzini, Ulli Samuelsson, Steven M. Schmidt, Susanne Iwarsson, Sofi Fristedt

**Affiliations:** 1 Department of Health Sciences, Lund University, Sweden; 2 Department of Engineering Technology, Technical University of Denmark, Denmark; 3 School of Education and Communication, Jönköping University, Sweden; 4 School of Health and Welfare, Jönköping University, Sweden; UNICATT: Universita Cattolica del Sacro Cuore, ITALY

## Abstract

Welfare technology (WT) use is increasingly advocated to facilitate aging in place. However, it remains unclear how older adults and homecare staff perceive these digital technologies in practice. This qualitative study investigated the ways in which current WT either facilitated or fell short in supporting older adults in their daily lives and homecare staff at work. Four key themes were developed using thematic analysis: 1) Successes and challenges with ease of use (i.e., how simple it is to interact with the WT); 2) Successes and challenges with usefulness (i.e., how effective the WT is in achieving its intended purpose); 3) Challenges with appearance, sounds and physical location; and 4) Challenges with technical maintenance and vulnerabilities. Findings revealed paradoxes in both older adult and homecare staff user perceptions. For instance, some successes with WT’s usefulness were highlighted, like GPS safety alarms in supporting older adult independence. However, challenges in usefulness were also exposed, like staff hiding “overused” safety alarms. Except for the medication robot, none of the WT devices clearly alleviated anticipated homecare staff shortages. WT perceived as auditorily or visually inadequate, that required more effort than users could comfortably manage, or that organizations could seamlessly integrate, were generally regarded as challenging. To overcome such challenges, greater involvement from older adults and homecare staff in the design and implementation of WT within homecare contexts is necessary. Refined criteria for prescribing WT to individuals, particularly regarding cognitive status, are additionally recommended by the researchers.

## Author summary

Digital technologies like welfare technology (WT) are increasingly viewed as a solution to help older adults live independently at home, as well as support homecare staff at work. However, research shows mixed results regarding how well current WT meets older adult and staff needs. We therefore chose to interview both staff and older adults about their perceptions of WT. Our study revealed that while some devices, like safety alarms with a GPS function, were viewed as supporting independence, many were perceived as challenging. Challenges arose due to issues with WT’s ease of use, usefulness and technical reliability. Older adults and homecare staff struggled with WT that was visually or audibly inadequate, and/or hard to integrate into care routines. Despite hopes that WT could address homecare staff shortages, most devices fell short on this ambition, apart from a robot that dispensed medication. Our study highlights the importance of involving older adults and caregivers in the design process of WT and calls for improved guidelines to match WT to individual needs, ensuring it supports users in meaningful ways.

## Introduction

Governments worldwide seek strategies to ensure a high quality of life as populations age [1]. To achieve this, high-income countries are emphasizing technology as a solution [2,3]. The Nordic region aims to be a leader in implementing welfare technology (WT)—digital solutions designed to enhance security, activity, participation, and independence for individuals with, or at risk of developing, disabilities—while also reducing reliance on caregivers [4,5]. WT also includes digital technology used by staff, such as applications that streamline routines and documentation [6].

WT implementation aligns with the goal of aging-in-place, that is, enabling older adults to continue residing in their “…own home and community safely, independently, and comfortably” [7]. While aging-in-place offers significant benefits, it also presents challenges and is sometimes criticized due to the extensive support it requires, which creates a growing and difficult-to-meet demand for home care services (HCS) provided by skilled professionals [8].

In Sweden, the context of the present study, public HCS are supported through government subsidies. Swedish HCS have a main objective of aiding older adults with daily tasks, fostering social interaction, and managing the distribution of medications [9]. The implementation of WT in HCS is part of a broader policy objective aimed at establishing the nation as a global leader in enhancing population welfare through digitalization [10]. The Swedish government expects digitalization to decrease the need for HCS staff recruitment through, for instance, the automation of care tasks [11]. Several of Sweden’s 290 municipalities have adopted the ambitious “digital first” slogan for their HCS, implying that primarily digital alternatives should be considered for care [12]. Decisions regarding how, when, and why WT is implemented are left to the discretion of individual municipalities; hence, adoption and utilization of WT vary across Sweden [11]. Older adults with declining function often stay in their homes for longer stretches of time than younger people, carrying out personal and instrumental activities of daily living, supported by staff [13–15]. HCS recipients are typically among the oldest old in Sweden, often living with severe co-morbidities [16]. This calls for an enhanced understanding of how WT, primarily used in the home, supports and challenges this more vulnerable group in their daily lives.

Previous research indicates that WT improves quality of life and independence for users as well as “saves time, costs, and personnel” within care organizations [16,17]. Night cameras, for example, allow older adults to sleep undisturbed, while simultaneously reducing the need for staff visits. However, a Swedish governmental investigation from 2024 [18] noted that current WT fails to meet user needs – though mostly organizational perspectives, rather than individual, were outlined. Studies focusing on digital technologies or WT recurrently lack participant involvement [19], or involve participant groups with insufficient diversity [20]. Previous studies on WT have applied models such as the Technology Acceptance Model (TAM), which explains technology adoption and use, primarily through the concepts of perceived usefulness and perceived ease of use [21–23]. Others have focused on developing alternative frameworks to understand technology acceptance [24]. However, as the perspective of TAM is that people should accept technology [19,25], studies taking the human rather than the technological perspective are warranted.

The current scientific literature does not comprehensively address the everyday successes and challenges associated with WT [15], that is, the positive outcomes or obstacles met during use. Previous literature has also shown that WT does not always reduce the workload of care workers [26] and that HCS staff have little influence over the types of WT they utilize [27]. As HCS staff perceive increasingly high demands, low control and low social support [28], integrating WT without their involvement can further increase work strain instead of giving intended benefits.

Investigating user perspectives is furthermore vital to assess whether the high policy ambitions for WT implementation align with the reality on the ground [3,13–15]. Whether, or to what extent, WT enhances older adults’ security, independence, and activity remains unclear, as does whether WT addresses HCS organizational needs, such as reducing reliance on caregivers. WT is an increasingly integral part of the HCS environment; however, research on user perceptions has yet to fully explore the ways in which its two primary user groups perceive these technologies [20,29–34]. Hence, the aim of this study was to explore how current WT challenges and/or succeeds to support older adults in their daily lives, as well as their HCS staff in their work.

## Methods

This was an experiential qualitative study [35] approved by the Swedish Ethical Review Authority, diary number 2022-01673-01.

### Recruitment and participants

We recruited HCS staff and community-dwelling older adults utilizing HCS from five Swedish municipalities. The purposive sampling strove for a variation in gender, age, and experience with WT. The municipalities represented large, mid-sized and more rural municipalities, selected based on geographic, socioeconomic, cultural, and ethnic diversity, as well as varying HCS systems and WT selections. Participant recruitment was supported by a contact person in each municipality.

HCS staff having worked in the municipality for at least six months received information about the study from the contact person. Those interested in taking part and consented to contact were called by the researchers. HCS staff then identified potential adult participants aged 65 years or older who used HCS and had WT. Older adults with known cognitive limitations that could influence their ability to provide informed consent were not considered. Older adults who had given permission to staff were contacted by one researcher via telephone, who further described the study. Interested participants were then booked for an interview.

In total, 26 older adults (women N = 17, mean age = 82 years, range = 65–99) and 26 HCS staff (women N = 17, mean age = 44, range = 22–65) volunteered to participate, and provided written informed consent. A translated and revised version of the University of California, San Diego Brief Assessment of Capacity to Consent [36] was used to strengthen the older adults’ process of informed consent. More than half of the older adults received HCS daily and lived alone in apartments (N = 18). Most staff had been working in HCS for a decade or more.

### Data collection

Data was collected through individual face-to-face interviews, based on an interview guide. Initially, two pilot interviews with one older adult and one HCS staff were conducted, and minor clarifying amendments were implemented. Further adjustments to the guide were carried out during subsequent interviews as deemed appropriate [37].

Older adults were asked about the WT they had been prescribed, and HCS staff about the WT they used or encountered in their work. Both groups were asked questions regarding how WT was used in a typical day. Positive and negative perspectives connected to these WT were then pursued. A complete list of interview questions can be found in Appendix I.

Older adults were interviewed in their homes, while HCS staff were interviewed at local HCS offices. All interviews were audio recorded. Interviews lasted one hour on average, and were conducted between September to December, 2022. Audio recordings were transcribed verbatim.

### Data analysis

The transcribed interviews were analyzed using inductive thematic analysis based on a six-step process adapted from Braun and Clarke [37].

First author SAS familiarized herself with the data through in-depth readings of all 52 interviews. Initial descriptive codes were then generated by SAS, capturing information on how WT was perceived by users in their daily lives or work environments. In parallel, co-author GCL independently coded approximately one-third of the interviews, leading to further discussions concerning meanings and patterns within the data. These initial codes were grouped into early themes by integrating similar codes. The preliminary themes were structured around each specific WT. Then, the researchers aimed to connect the challenges and successes both within and across the two user groups, as well as their experiences with WT. Through an iterative revision process, four final themes were identified, capturing patterns that linked overarching perceptions of WT while ensuring narrative coherence and relevance. Theme development was also informed by the write-up of findings, a common practice interwoven throughout qualitative thematic analysis [38].

The four themes revolved around four WT in particular:

1. The digital safety alarm is a device worn on a necklace or bracelet while at home. When the user presses the alarm button, s/he obtains voice contact with HCS staff or an emergency response center, to receive assistance in case of falls or other emergencies. Some municipalities provide GPS alarms, allowing for use out of home.
2. Digital mobile applications allow HCS staff to access HCS recipient information and care plans on their professional smartphones. In some municipalities, HCS staff document specific work duties or medications. In combination with a device installed in the door of the older person’s home, staff lock and unlock the door using mobile phones.
3. The medicine robot is an automatic medication dispenser filled with small portion bags containing prescribed medication, placed at the older person’s home. It has an audio reminder, which announces when it is time to take the medication.
4. The digital stove guard is designed to automatically cut off the electricity supply when excessive heat is detected, preventing potential fires.

## Findings

While both older adults and HCS staff perceived successes with WT supporting their daily lives and work, these successes were closely tied to challenges as highlighted through four key themes: 1) Successes and challenges with ease of use (i.e., how simple and straightforward it is to immediately interact with the WT); 2) Successes and challenges with usefulness (i.e., how beneficial and effective the WT is in achieving its intended purpose); 3) Challenges with appearance, sounds and physical location; 4) Challenges with technical maintenance and vulnerabilities.

### Theme 1: Successes and challenges with ease of use

There were both successes and challenges with the ease of use of WT. Older adults found digital safety alarms easy to use because pressing a button was the only required action. HCS staff also generally agreed on the alarm’s ease of use:

HCS staff 1:” [The alarm] is really good, or rather, I have nothing negative to say about it, like it works, and we haven’t had any technical problems, as far as I can recall.

However, despite positive comments, the alarm button was often considered too sensitive, leading to accidental activations:

Older adult A: “I think [the alarm] has worked really well, I can’t say anything other than that. But at the beginning, for a while, it was very, it was very sensitive [...], and as soon as I bumped it, I set off the alarm. I told them [the HCS staff], ‘Now you reallyhave to change this, because this isn’t working.”

In contrast, some examples were given regarding challenges with the alarm button being too difficult to push:

HCS staff 2: “Just the other day, I asked a woman to press her emergency alarm… because she told me that she had called for help several times, but no one came, and she spoke to the receiver, but no one answered. [...] And it turned out that she couldn’t press it because it was too hard.”

According to some older adults and HCS staff, digital alarm use was also sometimes complicated by the involvement of an intermediary—an external company, often based in a distant city—who initially received and managed alarm calls before forwarding them. In municipalities using intermediaries, HCS staff, often working within a dedicated alarm team, had to ‘accept’ responsibility for specific alarms. One staff member described certain challenges:

HCS staff 3: “—first, [the intermediary] gets the [call], and then they have to send it to us. And if we say no and can’t [take it]... Then they have to call [HCS staff outside the alarm team], so... there can be many steps. Usually, we don’t say no, but sometimes we have to, because... you can only be in one place at a time. We’re a bit understaffed right now.”

Some older adults also stated that the alarm response would be improved if someone within their local HCS staff group received the calls, as they had more immediate knowledge, and could thus improve staff reaction to and handling of alarms.

Regarding digital mobile applications, HCS staff appreciated having a variety of work tools accessible on one mobile device, making it easy to find HCS recipients’ contact and personal information.

HCS staff 4: “I think it is great, it’s so nice, you have everything in one place on your mobile phone, phone numbers are easy to reach and [the HCS recipients’ personal information] is easy to find on the phone…”.

However, digital mobile applications presented challenges for HCS staff at times, as not all aspects of documentation were interconnected within the mobile applications, reducing ease of use. For some participants, documentation of completed tasks or medicine distribution involved using two or three different channels (e.g., mobile phones, paper lists, and stationary computers). As a result, HCS staff sometimes had to rely on their memory until they returned to the office and could finish documenting on a computer.

HCS staff overall found digital locks easy to use. They explained that it was easy to knock on the older adult’s door and activate the lock receiver from the inside, creating a “very user-friendly” (HCS staff 5) device. However, the digital lock was not free from challenges. HCS staff remarked that digital locks did not function optimally with older, heavier doors. Another challenge commented on by several HCS staff was that the digital locks tended to “freeze up” or react too slowly when operated:

HCS staff 5: “[...] it is very, very slow, I would say. Sometimes you have to knock several times [to activate the technology]; something needs to be done about it, I feel.”

HCS staff 6: “It is very convenient when it works, but then sometimes there are issues... we might not be able to get into [the digital lock application] at all, and then we have to go and fetch physical keys from a central key cabinet.”

Though older adults did not utilize digital locks themselves, with one woman remarking: *“…* [digital locks] are their (the staff’s) kingdom*”* (older adult B), staff difficulties with the technology were noted. Older adults speculated that staff issues with locks were due to external factors like weather conditions, varying levels of technical proficiency among staff, or the general sluggishness of technological applications.

Concerning the medicine robot, though HCS staff considered this WT to be easy to use for older adults due to its large screen, inserting and extracting medication rolls was often difficult, negatively impacting ease of use for staff:

HCS staff 7: “Putting in the medication, it can take forever. If the person is going away on a trip... it can also take forever [to extract the medicine]....Because the medicine robot has to think and it has to ponder and... goodness, yes, it has to process and then it has to think again, and it can take forever. [...] Now when I was trying to get out medicine for a whole week, I got so irritated that I just pulled everything out, I said ‘Here you go! Take this now. I’ve been here for over half an hour.’

Moreover, HCS staff commented that the medicine robot required a certain level of cognitive ability from the older adult for optimal ease of use:

HCS staff 6: “[…] firstly, she didn’t understand when [the medicine robot] beeped that it was the robot beeping and that she should take her medicine, so she didn’t understand what to do. The few times she did understand she should take the medicine, the bag opened when it came out, and then she dropped the medicine instead […]. So, I think these robots are really great if you just need that reminder like ‘oh right’. But it also depends a bit on where you are cognitively, whether or not you understand.”

HCS staff furthermore found that stove guards led to challenges when preparing meals for older adults. Staff noted that stove guards often turned off unexpectedly and could be difficult to disable, posing challenges when cooking and older adults not receiving their planned meal:

HCS staff 2:”Once, I was using all four stove burners…and finally, I removed the battery from this stove guard... but I still couldn’t get the burner to start. So instead of being able to give her the meal I planned, I had to microwave brown beans and give her that without the pork.

Though installation routines claimed that user instructions should be displayed nearby, staff described not being able to locate them, decreasing ease of use:

HCS staff 8: No, I mean, you don’t see [instructions] often.

Interviewer: Okay. So you just have to...just try to use it?

HCS staff 8: Yes**…**If the stove is on without anything on the burner, it starts beeping, and if it continues for too long, it shuts off the stove. So it’s not so bad if you’re there, because then you hear it and can put something on the burner. But if it has been triggered on its own, then you have to reset the system, and sometimes it’s been completely impossible to do that… a few times, I’ve had to pull out the stove to unplug it and plug it back in again.

Though digital stove guards are designed to enhance safety, the challenges in ease of use sometimes overshadowed this benefit according to HCS staff.

### Theme 2: Challenges and successes with usefulness

The usefulness of WT was a second central issue raised by both older adults and HCS staff. The digital safety alarms were perceived as useful because they provided a sense of security for older adults, who knew they could easily call for assistance if needed:

Interviewer: “So out of all the technical devices […], which would you say matters most to you…?

Older adult B: “It was to be the alarm […] because you can get help with just about anything through it”.

Older adult C: “[The alarm’s] really a security. I don’t want to be without it because I know I can fall and not be able to get up.”

Recent advancements in WT, such as safety alarms with GPS functionality, also successfully enhanced user independence according to HCS staff as well as older adults:

Older adult D:” I can be wherever I want in the city and press the button, and they can tell exactly where I am.”

HCS staff additionally noted the alarm’s usefulness in that it allowed older adults to remain living in their own homes for longer. According to staff, many older adults expressed a preference for remaining at home, and given the low availability of residential care placements, HCS staff saw the alarm system as a compromise for both older adults and care providers.

Nevertheless, the alarm did not always offer older adults the same feeling of security as a physical person. One older adult explained that although she preferred to wash herself independently, she would not enter her shower without HCS staff physically present. Despite having a safety alarm in the shower, her fear of falling—especially while performing tasks like washing feet—was too great to rely solely on the alarm.

Some older adults moreover chose to discontinue their alarm service or not utilize it extensively because they viewed their mobile phone as more useful regarding security. HCS staff commented similarly on this issue:

Interviewer: “For what reasons do people stop using [the safety alarm]?

HCS staff 9: “… that can happen if someone realizes, ‘I haven’t used this for several months or even a year, and I feel secure with my mobile phone,’ and then they cancel it.”

Moreover, HCS staff reported challenges with older adult alarm users who had cognitive limitations, as some would press the safety alarm almost continuously, making this WT less useful both for themselves and the responding staff:

HCS staff 8:” “We’ve had people who have pressed the alarm maybe over 100 times in a day...We’ve had people who have pressed it while we were there... There’s always someone like that.”

As a provisional fix, one HCS staff described colleagues who hid the alarm button under the older adults’ wrist, creating a challenging, potentially dangerous situation in which the older adult would not be able to call for help if needed. Some older adults furthermore noted the impossibility for safety alarms to assist an unconscious person, and the potential dangers of this.

Digital locks were perceived as useful by HCS staff, as they reduced the physical strain of carrying multiple keys and coordinating key exchanges with colleagues:

HCS staff 10: “ The first thing is... you don’t have to carry around a hundred keys in your pocket. And the second thing is... you’re not afraid of losing those keys, because you’re human and you lose things, including keys. [...]. So... it’s easy, I mean it’s much, much easier for us to go in and out [of the residence].

Staff also found the digital locks useful because they enabled simultaneous registration in a digital system, providing evidence of a completed home visit. This was especially useful when an older adult forgot if a visit occurred, and concerned family members called to inquire.

The medicine robot was also perceived as useful in reducing the HCS staffs’ physical workload, and freeing up time for other matters:

HCS staff 7: “It’s one of those robots that dispenses medication. So we just go out and refill them every 14 days, so it’s very convenient. That way, we don’t have to go there three times just to distribute medication [...].”

The medicine robot moreover provided an additional safety check, as an alarm would be sent to a nurse if the dispensed medicine was left untouched. A similar function was found in the HCS staffs’ digital medicine delegation applications, which sent alarm notifications to a nurse if staff had not “signed” a medication distribution within the designated time window. However, in many instances this was a less useful feature, incurring “false” alarms due to staff simply forgetting to sign.

### Theme 3: Challenges with appearance, sounds and physical location

Both aesthetic and auditory aspects of WT could be perceived and reported as challenges. The physical placement of wearable devices also emerged as a challenge.

Firstly, the physical placement of the safety alarm on the body could create challenging or even life-threatening situations when an older adult could not reach it:

HCS staff 6: “We’ve also experienced a situation where a user fell and was lying down. He had the alarm on his wrist in a bracelet, but his arm was pinned underneath him so he couldn’t activate the alarm. He ended up lying there for several hours.”

Another challenge was that the safety alarm did not notify staff if a person stopped using it as recommended, such as failing to wear it. Some older adults commented that they had not been physically wearing the safety alarm, but were told to begin using it appropriately:

Older adult E: “I don’t really like it much, so I used to have it lying around for a while, but then I got scolded by my son that - ‘You have to wear it around your neck.’”

Municipalities typically conducted regular alarm checks. However, municipal routines for verifying alarm functionality were often voice-only and could not visually confirm if the older adult was wearing the alarm on their physical body.

At times, the safety alarm receiver was physically placed too far away from the user within the home, making it challenging for older adults to hear HCS staff – or for the staff to hear them. Another challenge could arise if an older adult was too far from the receiver for the alarm to register a button press, which could result in a failure to call for assistance:

HCS staff 11: “… we say that it has to do with the building’s walls, because sometimes [the alarm] might work outside as well, and then you have to test this yourself. Sometimes when we install an alarm, we are both in the backyard and down in the basement to test and make sure it works. But otherwise, we can only guarantee that it works inside the residence.”

Sometimes older adults preferred having the safety alarm placed around the neck, both for the physical accessibility as well as to more easily hide it from others:

Older adult F: I think this is better …it’s not visible, it can hang here under my clothes, so it’s not visible.

[…]It’s safer to have it around my neck. […] Yes, I feel safer with it [there].“

Women seemed to be particularly concerned with the appearance of the safety alarm, and reported a reduced satisfaction with the alarm’s aesthetic design. Some women chose not to wear their alarms at all at certain times:

Older adult E:”Because you know... it’s not always, if I go shopping I don’t take it off. But if I’m going to visit a friend or something [...]. We have a meeting once a month, a book café, there are five of us. Sometimes I take it off, but sometimes I have long sleeves. I’m so silly, it’s unbelievable. Childish.

Contrasting this, some HCS staff mentioned potential issues related to an alarm button being too visually discreet:

HCS staff 12: “And now the alarms are darker colored. Before they were white with a red button. So now they are a bit more discreet in some ways. It’s not easy to see them now; people might mistake them for a watch and press or fiddle with them a bit.”

The aesthetic appearance of medicine robots was also sometimes experienced as a challenge, and HCS staff reported older adults negatively commenting on the robot’s large size at first sight. Despite initial negative reactions, staff were often able to persuade older adults to keep the medicine robot for a short period, identifying that most would become accustomed to it over time. However, even after becoming accustomed to it, the robot’s pre-recorded voice directives could provoke feelings of annoyance and resistance in older adults:

Older adult G: ”She’s (*the robot’s*) really nagging, it’s like a voice talking, and I know it by heart. [...] She says, ‘it’s time to take your medicine.’ And then, every time I take out this medicine, she tells me to take them out of the bag and take with a glass of water. And I’ve learned it by heart now after a few years, but I guess I have to accept it.”

Consequently, both the appearance and auditory experience of a WT could also present user challenges, along with its physical location.

### Theme 4: Challenges with technical maintenance and vulnerabilities

Certain WT devices generated challenges because of their ongoing need for technical maintenance, as well as regarding their inherent vulnerabilities. As mentioned in the second theme, safety alarms with GPS could enhance perceptions of independence for some older adults. However, GPS alarms required continuous charging to continue operating. HCS staff described forgetting to charge GPS-alarms and receiving last-minute calls about the need to charge a dying battery. If the GPS-alarm’s battery was depleted, older adults could not utilize it in an emergency. Routines to ensure this perpetual maintenance were not always followed:

HCS staff 13: “Yes, there’s a new type of alarm that has come out. It includes GPS, but also functions differently. It needs to be charged, and not all users can manage that on their own. [...] So, it falls on us to ensure it’s charged. This is a step that often gets overlooked.”

Moreover, municipal routines did not always clarify which staff members were responsible for locating older adults if they ventured outside their designated GPS alarm zones – creating potential scenarios in which staff would not know how to respond. Routine alarm checks also sometimes missed older adults without regular HCS visits, raising concerns about the possibility of overlooking non-functioning safety alarms:

HCS staff 14: We’ve instructed them to test their alarm once a month, and if they have home care, then they (the staff) can handle it. But sometimes we have to call them because they haven’t tested it, so then we call and test it. It’s those who don’t use it regularly, it’s easy to forget [them]“.

Maintenance updates for digital applications occurred in some municipalities every other month. This basic maintenance caused challenges because it disrupted work routines, sometimes requiring staff to revert back to using paper schedules or physical keys. Staff thus reported that the applications could not be fully relied upon to replace analogue routines, emphasizing the necessity of maintaining an analogue backup.

The existence of multiple documentation systems, as described in the first theme, could also result in challenges for staff when performing delegated tasks. For example, when an insulin prescription was changed for an HCS recipient, delays or errors in updating the multiple systems could occur, leading to outdated instructions on the mobile application and/or paper medicine list. If one staff member failed to mark insulin as administered on the mobile app, the next staff member may mistakenly administer another dose, with potentially fatal consequences.

Besides vulnerabilities due to system updates, technological vulnerabilities such as the risk of external breaches (e.g., hacking) to digital lock applications were acknowledged. This again reinforced the need to keep physical keys and never wholly rely on WT:

HCS staff 14: “We still have the old key hiding spots for now [outside older adults’ homes], and it seems like there might be a large key cabinet coming here [...], considering the whole world now with cyber attacks and everything.”

One older adult compared the reliability of physical keys to vulnerable digital lock systems, which could be hacked or otherwise dismantled:

Older adult F: “[...] I don’t trust that it will work, it’s so vulnerable. I mean, you can easily open with a physical key, but that digital system is so vulnerable. You see... how easy it is to infiltrate, sabotage, and destroy... in computer systems and all that, so no, I don’t like it.”

Moreover, some older adults highlighted a vulnerability of the medicine robot, noting how easily its directives could be bypassed, allowing users to effectively “cheat”:

Older adult H: “ There’s no guarantee that I take [the medicine]. I might just take it out and throw it straight into the trash. [...] It’s up to my discretion.”

The medicine robot’s need for Internet connection was also sometimes perceived as an inherent technical vulnerability:

HCS staff 7: “Yes, it was in the beginning when we got the [medicine robot] and it kept... kept losing connection all the time... ‘Ah, damn, what a hassle!’ They really need to stay connected [...]. Otherwise, it can mess up terribly.”

Technical maintenance and vulnerabilities therefore presented user challenges, either by challenging the direct experience of WT use, or by requiring amendments to certain HCS routines, which could be difficult to follow.

## Discussion

Older adults and HCS staff represent an expanding target group for WT, the implementation of which is intended to address organizational challenges in HCS, while promoting independence among older adults and enabling aging-in-place [7]. This study adds to the scientific knowledge on WT by exploring perceptions of successes and challenges in WT use among older adults and HCS staff. Our findings reveal that, despite some successes in ease of use and perceived usefulness, certain challenges remain in improving how these two primary user groups perceive WT.

Two major paradoxes in WT use were revealed in our findings. A defining characteristic of a paradox is “the existence of simultaneous opposite assumptions or statements […]. Something is both liked and disliked or advantageous and disadvantageous at the same time” [39, pp. 418]. The first paradox lies in our finding that initial acceptance and continued use of WT can persist despite users having negative or challenging perceptions of the technology. An older adult might find the “voice” of a medicine robot irritating but accept and continue to use it. It remains unclear whether this acceptance and use is driven by staff influence, the user’s reluctance to change an established routine, or other factors like older adults’ awareness of care providers’ organizational challenges [40,41]. What is evident, however, is that technology acceptance and prolonged use do not necessarily occur due to positive perceptions of WT, indicating inherent paradoxes in WT use. Instead, people continue to use WT despite negative perceptions – possibly also due to the absence of better alternatives, at least at the time of our data collection in the participating Swedish municipalities.

This paradox in user perspectives also operates in reverse, as illustrated by the second paradox: despite expressing generally positive views about safety alarms, older adults reported challenges, such as not feeling sufficiently secure to shower with the sole accompaniment of an alarm. Similarly, though generally positive in their perceptions of digital locks, HCS staff reported challenges with use, such as incompatibility with certain doors. These contradictions highlight the inherent dichotomy in technology use: WT (as with all technology) works well, and is useful – until it does not, or is not, and that WT (as all technology) is inherently flawed, prone to failures and malfunctions [42]. Contradictory perceptions of technology use are also highlighted in the expectation/disconfirmation model of satisfaction [39], which emphasizes how user relationships with technological products often involve both positive and negative emotions.

A study [17] involving municipal decision-makers, who were asked under what circumstances WT surpasses or proves more reliable than humans, found that participants perceived WT as effective in minimizing the likelihood of human error and delivering more accurate outcomes. Our findings deliver contrasting evidence, like digital medicine distributions not being properly signed by HCS staff; thus, accentuating inadequacies in reducing human error. Despite such paradoxes, technology is widely regarded as an effective tool for streamlining work processes and reducing human error and effort. This positive stance toward technology is also evident in the broader policy discourse surrounding WT in Sweden and other similar countries, which view these digital technologies as the key solution to population aging [17]. This discourse falls neatly under Peine and Neven’s interventionist logic [43,44] which criticizes the notion that technological innovations can “solve” the “problem” of aging populations. While new technologies may solve some existing issues, they inevitably introduce new problems and forms of failure. Such failures must be anticipated, in the best cases preempted or at least planned for [45].

Our findings additionally highlight limitations of current WT in HCS, particularly regarding the ability to replace care staff. Digital applications, the fastest growing WT incorporated into Swedish HCS [18], are expected to streamline certain work tasks, particularly related to documentation, and reducing staff strain. However, the WT highlighted in our study do not appear to reduce staff workload to the extent that fewer people are required. In fact, staff explicitly noted challenges responding to security alarms due to perceived understaffing. When integrating WT into existing HCS, staff may perceive an increased workload, such as managing multiple digital and analog tools or charging GPS alarms—tasks that further technological training alone cannot alleviate. Recent studies [45,46] highlighted similar integration challenges, emphasizing the need for organizations to more carefully consider the prerequisites for practical incorporation of WT.

The medicine robot is one exception. Staff did in fact perceive a decreased need for visits solely dedicated to medication distribution thanks to the medicine robot, suggesting it did alleviate some of their workload. However, staff were still expected to manage the robot’s medicine refills and extractions. Widespread adoption of WT like the medicine robot furthermore risks exacerbating loneliness, as staff visits could represent HCS recipients’ sole opportunity for human interaction [47]. Loneliness among older adults presents a challenge when aiming to replace staff with technology, potentially exacerbating issues related to dehumanization and “cold care” [48–50]. Nevertheless, given predictions that Sweden will face a deficit of 65,000 care employees in just six years, innovative staff substitutions are likely necessary [50]. Similar alarming shortages are predicted on a global scale [51]. As stated, the four WT focused on in our study do not present a definitive solution for staff shortages – and nor do they seem to improve the psychosocial HCS work environment. For example, digital applications have been reported to pressure staff to rush visits due to minute-based schedules and monitoring systems [28,52,53].

Our findings moreover cast doubt on the ability of the safety alarm—the most commonly used WT [54]—to universally support aging-in-place, in particular for older adults facing cognitive decline, who were reported to use alarms more often and ineffectively. This critique was reflected in a recent article [55], which suggests that additional and/or novel types of technologies may be necessary to counteract challenges related to cognitive decline. Like the safety alarm, the usefulness of medicine robots was affected by the cognitive abilities of the user. This highlights the need for further developments in WT innovation and design, as well as the importance for supplying organizations (e.g., municipalities) to modify WT distribution according to changing needs – particularly cognition.

Our findings further point to the necessity of considering multiple user perspectives in WT design. For example, though digital stove guards are primarily installed to prevent fires due to unattended cooking by older adults, the WT could result in an older adult not receiving his/her expected meal due to challenges with ease of use for staff. Similarly, digital locks installed for staff convenience affect older adults, who rely on the proper functioning of these locks to receive their HCS visits. It is thus vital to be aware that WT are most likely encountered and handled by a variety of users, and that technology design should account for multiple and heterogeneous user groups [56]. Successes in usefulness and ease of use for one group, like older adults, do not always translate to success for another, like staff – highlighting that a foundational flexibility in WT design is imperative to meet the needs of multiple groups.

Our findings support that perceiving a WT as useful and easy to use leads to greater acceptance, both in line with TAM [21–23] as well as echoing previous research on digital technologies primarily intended for in-home use [57,58]. However, though perceived as both easy to use and useful, the safety alarm led some older adults, primarily women, to temporarily remove it to avoid embarrassment when meeting friends. Dissatisfaction with its stigmatizing appearance sometimes outweighs personal safety concerns, which is a troubling issue given its extensive use [54]. Zander et al. [3] stressed the need to reduce the underlying stigma of being a WT user – a challenge not explicitly identified by our participants but nonetheless a potential factor when the safety alarm is not worn as recommended. The four WT we focused on, primarily compensated for age-related decline— following a gerontechnological design approach dating back to the 1980s [43]. Broadening the function of WT could make the appearance and purpose of products less stigmatizing. Yang et al.‘s [59] model integrating TAM and Maslow’s Hierarchy of Needs suggested that individuals often prioritize higher-level desires over basic needs like safety when adopting technologies. Similarly, aesthetically pleasing design has been found to enhance perceived technology usability for older adults [60]. Therefore, WT designers should more carefully consider how to create more engaging and aesthetically pleasing designs, moving beyond compensatory functions. For example, modeling WT after familiar technologies like smartphones could lead to reduced stigma [43].

In short, our findings indicate that individual and organizational needs must still be better considered in relation to WT development and implementation. A recent Swedish report [18] explains some of these enduring issues, stating that municipalities commonly prioritize long-term goals in relation to WT rather than concrete plans, and they likely overestimate their analyses of target group needs. Refining the criteria for prescribing WT and creating a detailed plan for its integration into existing organizational procedures is essential, and currently a process reported to be somewhat erratic [61]. Our stance is thus consistent with scholars calling for more innovative approaches in which older adults and HCS staff have committed roles in designing and situating technologies within their relevant contexts [16,17]. WT that is incorrectly prescribed or demands more than a user or an organization can easily manage can become not only unsuccessful, but a potential danger.

Based on our findings, we argue that end users must be genuine partners in the process of WT design and implementation if the perceptions of these devices are to be improved going forward. Perhaps some of the paradoxical challenges we highlight could be mitigated using Melles et al.’s [62] human-centered approach, which considers both individual and organizational needs, aiming to “understand people and solve the right problems”. Such an approach intends to ensure that users receive WT that best facilitates their daily needs. Collaborative involvement better assures that the conceptual design, development and implementation of WT is attuned to practical needs, fostering more meaningful use for all stakeholders.

### Methodological considerations

The Standards for Reporting Qualitative Research [63] consists of 21 items, all of which have been considered in the analysis and write up of this research. The qualitative approach centered on amplifying the voices of those most directly impacted by WT, reinforcing the study’s credibility in line with Lincoln and Guba’s trustworthiness criteria [64]. The inclusion of a substantial number of interviews, surpassing that of most qualitative studies, and the involvement of an interdisciplinary research team further strengthened trustworthiness.

Braun and Clarke have taken a strong stance against the concept of data saturation and stated that “A broader [study] scope requires more participants generally” as well as “What you want to make sure is that you have enough data to tell a rich story, but not too much that it precludes deep, complex engagement with the data in the time available.” [35, pp. 23]. Consequently, our interview recruitment aimed to capture user perceptions from a reasonable number of participants in each municipality, while also reflecting the distinct HCS systems, WT selections, and population demographics of the five municipalities. However, while the HCS staff participants were demographically diverse, the older adults were not as ethnically diverse as we desired, implying that the findings to a lesser extent reflect the perspectives of marginalized populations of older adults in Sweden.

It should be noted that we decided to include only four types of WT to achieve narrative cohesiveness. Consequently, perceptions of other WT were not given space in the findings, potentially discounting other successes and challenges with WT use. In addition, HCS staff provided richer interview data, as they had often experienced a broader range of WT cases, given their work with numerous individuals and their own technological tools. In contrast, older adult participants could only draw from their personal experiences. Therefore, a larger number of quotations from HCS staff are found in our findings.

## Conclusion

This study shows that although WT offers potential benefits for older adults and HCS staff, they bring unforeseen issues and potential failures that require foresight, proactive measures, and strategic planning. The findings highlight a variety of paradoxes in which WT perceived positively are reported to have challenges, but WT perceived more negatively still become accepted and used. Apart from the medicine robot, there were no obvious examples displaying how WT would effectively ameliorate predicted staff shortages. In contrast, in some cases WT creates additional work for staff. WT that demands more than what a user or an organization can easily manage are perceived as challenging and/or potential dangers. Thus, design and implementation strategies affording older adults and HCS staff more substantial roles in situating WT within homecare contexts are called for. Refined criteria for prescribing WT to individuals are recommended by the researchers, along with improved conceptualization and design, particularly regarding users with cognitive limitations.

## Appendix I

### Interview questions

- What type or types of welfare technology do you use in your daily life or work?
- How is welfare technology used in your daily life or work?
- Do you feel that welfare technology fulfils its purpose?
- What is your attitude towards welfare technology in general?
- Do you have access to the welfare technology you need in your daily life or work?
- Give me examples of when and how you use welfare technology during a typical day.
- What do you think makes a certain welfare technology give you a positive or negative experience?
- Do you feel that you have the knowledge needed to use welfare technology?
- Do you find that you use the welfare technology in unexpected ways?

## References

[1] Hsu M, Yamada T. Population aging health care and fiscal policy reform The challenges for Japan. Scand J Econ. 2019;121(2):547–77. doi: 10.1111/sjoe.12280

[2] Woll A. Use of welfare technology in elderly care [Doctoral thesis]. Oslo: University of Oslo, Faculty of Mathematics and Natural Sciences; 2017. Available from: https://urn.nb.no/URN:NBN:no-xxxxxx

[3] Zander V, Johansson-Pajala RM, Gustafsson C. Methods to evaluate perspectives of safety, independence, activity, and participation in older persons using welfare technology: A systematic review. Disabil Rehabil Assist Technol. 2019;15(4):373–93. doi: 10.1080/17483107.2019.1574919 30786779

[4] Zander V, Gustafsson C, Landerdahl Stridsberg S, Borg J. Implementation of welfare technology: a systematic review of barriers and facilitators. Disabil Rehabil Assist Technol. 2023; 18(6):913–28. doi: 10.1080/17483107.2021.1938707 34129802

[5] Socialstyrelsen. E-hälsa och välfärdsteknik i kommunerna 2019 [E-health and welfare technology in municipalities 2019]. Socialstyrelsen; 2019. Available from: *https://www.socialstyrelsen.se/globalassets/sharepoint-dokument/artikelkatalog/ovrigt/2019-5-10.pdf*

[6] Modig A. Välfärdsteknologi inom äldreomsorgen: En kartläggning av samtliga Sveriges kommuner [Welfare technology in elderly care: A survey of all municipalities in Sweden]. Hjälpmedelsinstitutet; 2012.

[7] American Planning Association, National Association of County and City Health Officials. Healthy places terminology. Centers for Disease Control and Prevention; 2009.

[8] Hjelle KM, Tuntland H, Førland O, Alvsvåg H. Driving forces for home-based reablement: A qualitative study of older adults’ experiences. Health Soc Care Community. 2016;25(5):1581–9. doi: 10.1111/hsc.12324 26806390

[9] Assander S, Bergström A, Olt H, Guidetti S, Boström A-M. Individual and organisational factors in the psychosocial work environment are associated with home care staff’s job strain: A Swedish cross-sectional study. BMC Health Serv Res. 2022;22:1418. doi: 10.1186/s12913-022-08699 36434716 10.1186/s12913-022-08699-4 PMC9701045

[10] The Swedish eHealth Agency. Vision e-hälsa 2025: Gemensamma utgångspunkter för digitalisering i socialtjänst och hälso- och sjukvård [Vision e-health 2025: Common starting points for digitization in social services and healthcare]. The Swedish eHealth Agency; 2022. Available from: *https://ehalsomyndigheten.se/uppfoljning-vision-e-halsa-2025-rapport-avseende-2021.pdf*

[11] Samén A, Lindberg J, Andersson K. Disembodied care: Articulations of care in municipal policy regarding welfare technologies in eldercare. Nordic J Soc Res. 2024;15(1):1–14. doi: 10.18261/njsr.15.1.1

[12] Chatterjee S, Chowdhury AR. Elderly health and wellness: A review of global policies and frameworks. Glob Public Health. 2022; 17(4):478–92. 10.1080/17441692.2021.1990312

[13] Luoma-Halkola H, Häikiö L. Independent living with mobility restrictions: Older people’s perceptions of their out-of-home mobility. Ageing Soc. 2022;42(2):249–70. doi: 10.1017/S0144686X20000823

[14] Kim KT, Hawkins BA, Lee YH, Kim H. Social support and daily life activity: Determinants of aging well. Act Adapt Aging. 2023;47(2):171–94. doi: 10.1080/01924788.2022.21060

[15] Lawton MP. Aging and performance of home tasks. Hum Factors. 1990;32(5):527–36. doi: 10.1177/001872089003200503 2074107

[16] Brändström A, Meyer AC, Modig K, Sandström G. Determinants of home care utilization among the Swedish old: nationwide register-based study. Eur J Ageing. 2022;19:651–62. doi: 10.1007/s10433-021-00669-9 36052192 PMC9424454

[17] Nordby H, Eriksson S. Ethical dilemmas in the use of welfare technology in eldercare: A systematic literature review. Nurs Ethics. 2021; 28(6):870–84. doi: 10.1177/0969733021996416

[18] Nguyen MH, Dinh T. Digital inclusion among the elderly: Facilitators and barriers. J Gerontol Soc Work. 2023; 66(3): 1–17. doi: 10.1080/01634372.2022.211570336573260

[19] Swedborg A. Welfare technologies for older adults: Policy implications for ethical decision-making. J Appl Ethics. 2021;38(2):110–23.

[20] European Commission . The Silver Economy: A study on the aging population and innovation. European Union Publications Office; 2022. Available from: https://ec.europa.eu/research/social-sciences/silver-economy-study_en.pdf

[21] Chen K, Chan AHS. Gerontechnology acceptance by elderly Hong Kong Chinese: a senior technology acceptance model (STAM). Ergonomics. 2014;57(5):635–52. doi: 10.1080/00140139.2014.895855 24655221

[22] Davis FD. Perceived usefulness, perceived ease of use, and user acceptance of information technology. MIS Quarterly. 1989;13(3):319–340. doi: 10.2307/249008

[23] Harris MT, Rogers WA. Developing a Healthcare Technology Acceptance Model (H-TAM) for Older Adults with Hypertension. Ageing Soc. 2023;43(4):814–34. doi: 10.1017/S0144686X21001069 37007645 PMC10062492

[24] Mahmood A, Yamamoto T, Lee M, Steggell C. Perceptions and use of gerotechnology: implications for aging in place. J Hous Elderly. 2008;22(1–2):104–26. doi: 10.1080/02763890802097144

[25] Andrich R, Mathiassen N-E, Hoogerwerf E-J, Gelderblom GJ. Service delivery systems for assistive technology in Europe: An AAATE/EASTIN position paper. Technol Disabil. 2013;25(3):127–46. doi: 10.3233/TAD-130381

[26] Lindeman DA, Kim KK, Gladstone C, Apesoa-Varano EC. Technology and caregiving: emerging interventions and directions for research. Gerontologist. 2020;60(Suppl 1):S41–S49. doi: 10.1093/geront/gnz178 32057082 PMC7019659

[27] Lydahl D. Välfärdsteknikens värden – en delrapport [The values of welfare technology – a partial report]. FoU i Väst, Göteborgsregionen; 2021. Available from: https://goteborgsregionen.se/download/

[28] Aronsson G, Marklund S, Leineweber C, Helgesson M. The changing nature of work - Job strain, job support and sickness absence among care workers and in other occupations in Sweden 1991-2013. SSM Popul Health. 2021;15:100893. doi: 10.1016/j.ssmph.2021.100893 ; PMCID:34522762 PMC8426264

[29] Myndigheten för vård- och omsorgsanalys. Digital teknik med äldre i fokus: en delredovisning av utvärderingen av överenskommelsen om digitaliseringen i äldreomsorgen [Digital technology with a focus on older adults: a partial report of the evaluation of the agreement on digitization in elderly care]. Stockholm: Myndigheten för vård- och omsorgsanalys; 2021. PM 2021:2. Available from: *https://www.vardanalys.se/rapporter/digital-teknik-med-aldre-i-fokus/*

[30] Russo N, Eriksson J, Christensen J. Target framework for sustainable welfare technology deployment in eldercare. UKAIS Conf Proc. 2021:507–16.

[31] Sallinen M, Hentonen O, Teeri S. Ethical dilemmas related to the use of safety technology in service house environments. Scand J Caring Sci. 2020;34(1):199–205. doi: 10.1111/scs.1272 31250937

[32] Östlund B. Digitalization of later life: What prevents the care sector from meeting the rapid digitalization of older populations? In: Kalra J, Lightner NJ, Taiar R, editors. Advances in Human Factors and Ergonomics in Healthcare and Medical Devices. AHFE 2021. Lecture Notes in Networks and Systems. Vol 263. 2021. p. 287–298. doi: 10.1007/978-3-030-80744-3_3

[33] Karlsen C, Ludvigsen MS, Moe CE, Haraldstad K, Thygesen E. Experiences of community-dwelling older adults with the use of telecare in home care services: A qualitative systematic review. JBI Database System Rev Implement Rep. 2017;15(12):2913–80. doi: 10.11124/JBISRIR-2017-00334 29219874

[34] Malmgren Fänge A, Carlsson G, Chiatti C, Lethin C. Using sensor-based technology for safety and independence - the experiences of people with dementia and their families. Scand J Caring Sci. 2020;34(3):648–57. doi: 10.1111/scs.1276 31614031

[35] Braun V, Clarke V. Successful qualitative research: A practical guide for beginners. London: SAGE Publications; 2013.

[36] Jeste DV, Palmer BW, Appelbaum PS, Golshan S, Glorioso D, Dunn LB, et al. A new brief instrument for assessing decisional capacity for clinical research. Arch Gen Psychiatry. 2007;64(8):966–74. doi: 10.1001/archpsyc.64.8.966 17679641

[37] Braun V, Clarke V. Thematic analysis: A practical guide. London: SAGE Publications; 2021.

[38] Byrne D. A worked example of Braun and Clarke’s approach to reflexive thematic analysis. Qual Quant. 2022;56:1391–412. doi: 10.1007/s11135-021-01182-y

[39] Johnson D, Bardhi F, Dunn DT. Understanding how technology paradoxes affect customer satisfaction with self-service technology: The role of performance ambiguity and trust in technology. Psychol Mark. 2008;25(5):416–43.

[40] Aronson J. Silenced complaints, suppressed expectations: The cumulative effects of home care rationing. Int J Health Serv. 2006;36(3):535–56. 16981630 10.2190/CGPJ-PRWN-B1H6-YVJB

[41] Westerberg K, Hjelte J, Josefsson S. Understanding eldercare users’ views on quality of care and strategies for dealing with problems in Swedish home help services. Health Soc Care Community. 2017;25(2):621–9. doi: 10.1111/hsc.12351 27109545

[42] Sundén J. Glitch, genus, tillfälligt avbrott: Femininitet som trasighetens teknologi [Glitch, gender, temporary interruption: Femininity as the technology of brokenness]. Lambda Nordica. 2016;(1-2):23–45. Available from: https://search.ebscohost.com/login.aspx?direct=true&AuthType=ip,uid&db=edsswe&AN=edsswe.oai.DiVA.org.sh.31227&site=eds-live&scope=site Accessed November 11, 2024

[43] Peine A, Neven L. From Intervention to Co-constitution: New Directions in Theorizing about Aging and Technology. Gerontologist. 2019 Feb;59(1):15–21. doi: 10.1093/geront/gny050 29850812

[44] Taylor L. There is an app for that: Technological solutionism as COVID-19 policy in the Global North. The New Common. 2021 Mar 20:209–15. doi: 10.1007/978-3-030-65355-2_30 PMCID: PMC7978704

[45] Brandenberger IA, Hasu MA, Nerland M. Integrating technology with work practices in primary care: challenges to sustainable organizing “from within”. Learn Organ. 2024;31(3):317–36. doi: 10.1108/TLO-01-2023-0009

[46] Mohammadnejad F, Freeman S, Klassen-Ross T, Hemingway D, Banner D. Impacts of Technology Use on the Workload of Registered Nurses: A Scoping Review. J Rehabil Assist Technol Eng. 2023;10:. doi: 10.1177/20556683231180189 37342268 PMC10278405

[47] Mertz L, Tornbjerg K, Nøhr C. User perception of automated dose-dispensed medicine in home care: A scoping review. Healthcare (Basel). 2021;9(10):1381. doi: 10.3390/healthcare9101381 34683061 PMC8544441

[48] Kim K, Gollamudi SS, Steinhubl S. Digital technology to enable aging in place. Exp Gerontol. 2017;88:25–31. doi: 10.1016/j.exger.2016.11.013 28025126

[49] Long KM, Casey K, Bhar S, Al Mahmud A, Curran SPN, Hunter K, et al. Understanding perspectives of older adults on the role of technology in the wider context of their social relationships. Ageing Soc. 2024;44(6):1453–76. doi: 10.1017/S0144686X2200085X

[50] Stahl BC, Coeckelbergh M. Ethics of healthcare robotics: Towards responsible research and innovation. Robot Auton Syst. 2016;86:152–61. doi: 10.1016/j.robot.2016.08.018

[51] World Health Organization. Global strategy on human resources for health: Workforce 2030. Geneva: World Health Organization; 2016. Available from: https://www.who.int/publications/i/item/9789241511131 OrganizationGeneva2016 https://www.who.int/publications/i/item/9789241511131Available from:

[52] Hofmann B. Ethical challenges with welfare technology: a review of the literature. Sci Eng Ethics. 2013;19(2):389–406. 10.1007/s11948-011-9348-1. Epub 2012 Jan 5. 22218998

[53] Svärdh SA, Lorenzini GC, Siverskog A, Schmidt SM, Iwarsson S, Fristedt S. Detangling experiences of agency in welfare technology use by home care recipients and their staff. Disabil Rehabil Assist Technol. 2024 Dec 11:1–13. doi: 10.1080/17483107.2024.2435566 39661532

[54] Socialstyrelsen. Trygghetslarm vanligaste första stöd för äldre [Safety alarms most common initial support for the elderly] [Internet]. 2016 Jun 21 [cited 2025 Jan 9]. Available from: https://www.socialstyrelsen.se/om-socialstyrelsen/pressrum/press/trygghetslarm-vanligaste-forsta-stod-for-aldre

[55] Puaschitz NGS, Jacobsen FF, Berge LI, Husebo BS. Access to, use of, and experiences with social alarms in home-living people with dementia: results from the LIVE@Home.Path trial. Front Aging Neurosci. 2023 May 22;15:1167616. doi: 10.3389/fnagi.2023.1167616 ; PMCID: PMC10239917 37284020 PMC10239917

[56] Oudshoorn N, Pinch T, editors. How users matter: the co-construction of users and technology. Cambridge (MA): MIT Press; 2003.

[57] Žvanut B, Mihelič A. Qualitative study on domestic social robot adoption and associated security concerns among older adults in Slovenia. Front Psychol. 2024 Jan 25;15:1343077. doi: 10.3389/fpsyg.2024.1343077 ; PMCID: PMC10850379. 38333061 PMC10850379

[58] Anttila M, Koivisto J, Luoma M-L, Anttila H. How to adopt technologies in home care: a mixed methods study on user experiences and change of home care in Finland. BMC Health Serv Res. 2023;23:1342. doi: 10.1186/s12913-023-10368-z 38042800 PMC10693073

[59] Yang Y, Yu X, Zhang Z, Gan L. Integrating Technology Acceptance Model With Maslow’s Hierarchy Needs Theory to Investigate Smart Homes Adoption. IEEE Access. 2023;11:75078–75093. doi: 10.1109/ACCESS.2023.3300724

[60] Bao H, Lee EWJ. Examining the antecedents and health outcomes of health apps and wearables use: an integration of the technology acceptance model and communication inequality. Behav Inf Technol. 2024;43(4):695–716. doi: 10.1080/0144929X.2023.2183062

[61] Samhällskontraktet. Hälsa, välfärd och vård – Hem för vård och hälsa: Slutrapport för delprojekt 3D [Health, welfare, and care – Homes for care and health: Final report for subproject 3D]. 2021. Available from: https://www.samhallskontraktet.se/download/18.4a782d2d17c01ffabdd99b08/1633613765170/HV3D%20slutrapport.pdf

[62] Melles M, Albayrak A, Goossens R. Innovating health care: key characteristics of human-centered design. Int J Qual Health Care. 2021 Jan 12;33(Supplement_1):37–44. doi: 10.1093/intqhc/mzaa127 ; PMCID: PMC7802070. 33068104 PMC7802070

[63] Equator Network. SRQR: Standards for Reporting Qualitative Research [Internet]. 2021 [cited 2025 Jan 9]. Available from: https://www.equator-network.org/reporting-guidelines/srqr/

[64] Forero R, Nahidi S, De Costa J, Mohsin M, Fitzgerald G, Gibson N, et al. Application of four-dimension criteria to assess rigor of qualitative research in emergency medicine. BMC Health Serv Res. 2018;18(1):120. doi: 10.1186/s12913-018-2915-2 29454350 PMC5816375

