# Supplementary material for: “It is very convenient when it works – successes and challenges with welfare technology” - a qualitative study
Source: PLOS Digit Health. 2025 Apr 24;4(4):e0000844. doi: 10.1371/journal.pdig.0000844 (PMC12021168; doi:10.1371/journal.pdig.0000844)
Supplement: Attachment — Submitted filename: Rebuttal letter Svardh et al WT interview study.docx [file pdig.0000844.s003.docx]

**Rebuttal letter**

**'Response to Reviewers'**

Svärd et al, March 2025

**Review Comments to the Author**

Reviewer # 1 General:

**1. Reviewer comment:**

Reviewer #1: In my opinion the paper fits with PLOS requirements, I thin that the requirement "statistical analysis been performed appropriately and rigorously" isn't applicable because it is a qualitative reasearch and there aren't enough references to assess this point. In the introduction there is a light disclosure about the composition of the cluster of patients that seem to be representative of adults population but I don't think that the parameter of statistical analysis can be applied to this paper

**Author comment:**

This comment is correct. No statistical analysis was performed in our study. Qualitative methods were used to analyze the interview data.

Reviewer #2: General:

**2. Reviewer comment:**

Language revision is required. Sentences should be concise and to the point, authors tend to use long sentences which are -sometimes- confusing.

**Author response:**

Thank you. The manuscript text has been re-read and sentences have been simplified or edited where appropriate. An example of this editing is provided below:

*The original introduction initially began as follows:*

“Governments around the globe look for approaches to a brighter future, where a good quality of life is sustained when becoming older [1]. To achieve this, high-income countries are emphasizing technology as a solution [2, 3]. The Nordic region aspires to lead in implementing welfare technology (WT), that is, digital technologies designed to maintain or improve security, activity, participation, and independence for individuals who have, or are at increased risk of developing disability, thereby reducing reliance on caregivers [4,5]. WT also includes digital tools used by staff, such as applications that streamline routines and document tasks [6].

WT implementation aligns with the goal of aging-in-place, that is, enabling older adults to continue residing in their “[…] own home and community safely, independently, and comfortably” [7]. While beneficial, aging-in-place poses challenges and is sometime criticized because the comprehensive support required drives a growing demand for homecare services (HCS) provided by skilled professionals [8]. As many countries face a shortage of qualified HCS personnel to meet this demand, policymakers turn their hope to WT as a substitute and/or complement to traditional care services.”

*We have revised the first two paragraphs as follows:*

Governments worldwide seek strategies to ensure a high quality of life as populations age [1]. To achieve this, high-income countries are emphasizing technology as a solution [2, 3]. The Nordic region aims to be a leader in implementing welfare technology (WT)—digital solutions designed to enhance security, activity, participation, and independence for individuals with, or at risk of developing, disabilities—while also reducing reliance on caregivers [4,5]. WT also includes digital technology used by staff, such as applications that streamline routines and documentation [6].

WT implementation aligns with the goal of aging-in-place, that is, enabling older adults to continue residing in their “…own home and community safely, independently, and comfortably” [7]. While aging-in-place offers significant benefits, it also presents challenges and is sometimes criticized due to the extensive support it requires, which creates a growing and difficult-to-meet demand for home care services (HCS) provided by skilled professionals [8].

We have also made changes in the Discussion section. Please see lines 548-552, 565-571, and 582-585.

**Reviewer comment:**
The abbreviation HCS is written as HSC many times. Is this a typo or does it refer to another term? Please explain.

**Author response:**

Thanks a lot for notifying us about this error. Unfortunately, HSC was written several times, but has now been corrected. HCS is the correct abbreviation and stands for “home care services”.

**Reviewer comment:**

Title:
Title of the study is long and does not capture the study method and purpose. Please modify.

**Author response:**

The original title was “It is very convenient when it works – successes and challenges with welfare technology.” This fully complies with PLOS Digital Health guidelines regarding title length, which state that titles can contain a maximum of 200 characters. The title contained 92 characters. Given the qualitative study design, a title including a participant quote is relevant. Still, the title could contain more information about the method. The revised title reads as follows: **“It is very convenient when it works – successes and challenges with welfare technology” - a qualitative study.”**

**Reviewer comment:**

Abstract:
Avoid using abbreviations in the abstract section.

**Author response:**

Thank you for catching the mistake of using HCS without clarification. This has now been corrected in the abstract (see line 65).

**Reviewer comment:**
Introduction:
Page 8 line 96: I am not sure what the brackets here refer to.

**Author response:**

You are correct, the brackets do not comply with standard quoting conventions and have been removed.

**Reviewer comment:**
Themes:
I would not use the word "challenges" for every dissatisfying quality of WT. Authors should reconsider that label.

**Author response:**

After carefully considering this comment, we find it difficult to link unsatisfactory perceptions of WT use to a different overarching term. The term ‘challenges’ captures quite well how users sometimes perceived the technologies. In the Introduction, we write: “The current scientific literature does not comprehensively address the everyday successes and challenges associated with WT [15], that is, the positive outcomes or obstacles met during use” (lines 131-132). Accordingly, we defined challenges as “obstacles met during use”. This description adequately encapsulates the challenging issues we have highlighted regarding WT’s ease of use, usefulness, appearance, sounds and physical location as well as technical maintenance and vulnerabilities. Including the term ‘challenges’ in the theme also directly relates to the study aim, which is, to explore how current WT challenges and/or succeeds to support older adults in their daily lives, as well as their HCS staff in their work. Consequently, we decided to keep the terminology used in the original manuscript.

**Reviewer # 2 comments (made directly in manuscript)**

“when you use for the first time, you shoul write the whole name (Health care Service)”

**Author response:**

Thank you. You will find that the abbreviation is written out in the second paragraph of the introduction line 99, indicating that we mean “home care services”.

**Reviewer comment:**

“explain what is TAM”

**Author response:**

Thank you for this comment. We have now included a concise description of TAM, see lines 123-125:

“Previous studies on WT have applied models such as the Technology Acceptance Model (TAM), which explains technology adoption and use, primarily through the concepts of perceived usefulness and perceived ease of use [21-23].”

**Reviewer comment:**

“you should underline not olny the usefulness and preference but also the advantage of caring old people at their home both in term of costs for HCS and in term of benefits for the lives of old people that usually suffer the change of care setting fro home to hospital. In general the best benefit of WT (not limited to alarm but for istance telemonitoring device, IoT device) is to avoid to change people' habits beacuse they can remain at home instead of asking them to make several travels for hospital visits and in the worst case hospitalisation to be constantly monitored. I think you should underline more this advantage”

**Author response:**

Thank you for your comment. Your reflections are interesting and relevant. However, the themes presented as findings from a qualitative analysis should only convey the participants’ perceptions. Author reflections and discussion points regarding the findings come later in the Discussion.

Your comment about reducing care costs and increasing independence at home is nonetheless addressed in the Introduction as one of the perceived advantages of WT: “Previous research indicates that WT improves quality of life and independence for users as well as “saves time, costs, and personnel” within care organizations [16, 17].” (lines 118-119). We also allude to these individual and organizational concerns in the Discussion several times, for instance when referencing the advantages of WT. However, we take a more critical stance on these issues: “This positive stance toward technology is also evident in the broader policy discourse surrounding WT in Sweden and other similar countries, which view these digital technologies as the key solution to population aging [17]. This discourse falls neatly under Peine and Neven’s interventionist logic [43, 44] which criticizes the notion that technological innovations can "solve" the "problem" of aging populations.” (lines 512-516)

Furthermore, in lines 95-99 of the Introduction, we write “WT implementation aligns with the goal of aging-in-place, that is, enabling older adults to continue residing in their “…own home and community safely, independently, and comfortably” [7]. While aging-in-place offers significant benefits, it also presents challenges and is sometimes criticized due to the extensive support it requires, which creates a growing and difficult-to-meet demand for home care services (HCS) provided by skilled professionals [8].”

To better emphasize the specific individual advantages of WT that you mentioned in your comment, we have now added a clarifying phrase in the first sentence of the Discussion: “Older adults and HCS staff represent an expanding target group for WT, the implementation of which is intended to address organizational challenges in HCS, while promoting independence among older adults and enabling aging-in-place [7].” (line 479)

**Reviewer comment:**

“have you provied a training or any type of education about the right use of the device? This could reduce the misuse of the device”

**Author response:**

The manuscript authors are researchers and not practitioners. Accordingly, we are not carrying out any form of training or studying interventions. However, we will be communicating our results to municipal service providers.

**Reviewer comment:**

“This is a predictable outcome because because a key point to a successfull use of WT it's a patient centric approach that involve patient since the creation of thedevice that must be design following a design thinking method so it reflect patients needs”

**Author response:**

Yes, we agree, and the following text from the Discussion section reflects the essence of your comment:

**“**Our stance is thus consistent with scholars calling for more innovative approaches in which older adults and HCS staff have committed roles in designing and situating technologies within their relevant contexts [16, 17]. WT that is incorrectly prescribed or demands more than a user or an organization can easily manage can become not only unsuccessful, but a potential danger. Based on our findings, we argue that end users must be genuine partners in the process of WT design and implementation if the perceptions of these devices are to be improved going forward. Perhaps some of the paradoxical challenges we highlight could be mitigated using Melles et al.’s [62] human-centered approach, which considers both individual and organizational needs, aiming to “understand people and solve the right problems”. Such an approach intends to ensure that users receive WT that best facilitates their daily needs.” (lines 587-597)

**Reviewer comment:**

“this underline the necessity of a training for the use of WT solutions both for staff and for patients to avoid misuse”

**Author comment:**

We are unsure about how further technological training is relevant in this particular part of the Discussion; however, we have included a reference to training based on the next reviewer comment.

**Reviewer comment:**

“this could be reduce with an adeguated training of staff and after a period of adaptation as happend everytimes we introduce a new practice, guidelines or tool. It's part of innovation not only in term of tools but it is related to each change”

**Author comment:**

Thank you for your comment. We see it as difficult for training to solve this challenge facing HCS staff. However, we have addressed this comment by editing the text as follows:

“When integrating WT into existing HCS, staff may perceive an increased workload, such as managing multiple digital and analog tools or charging GPS alarms—tasks that further technological training alone cannot alleviate.” (lines 525-528)
