# Supplementary material for: “It is very convenient when it works – successes and challenges with welfare technology” - a qualitative study
Source: PLOS Digit Health. 2025 Apr 24;4(4):e0000844. doi: 10.1371/journal.pdig.0000844 (PMC12021168; doi:10.1371/journal.pdig.0000844)
Supplement: Peer review history

# Author comment: Author response to Decision Letter 0

**DOI:** 10.1371/journal.pdig.0000844.r001

23 Jan 2025

# Peer review: Decision Letter 0

**DOI:** 10.1371/journal.pdig.0000844.r002

**Authors:** Francesco Andrea Causio

25 Feb 2025

PDIG-D-25-00045It is very convenient when it works” – successes and challenges with welfare technologyPLOS Digital Health Dear Dr. , Thank you for submitting your manuscript to PLOS Digital Health. After careful consideration, we feel that it has merit but does not fully meet PLOS Digital Health's publication criteria as it currently stands. Therefore, we invite you to submit a revised version of the manuscript that addresses the points raised during the review process. Please submit your revised manuscript within 30 days Mar 27 2025 11:59PM. If you will need more time than this to complete your revisions, please reply to this message or contact the journal office at digitalhealth@plos.org. When you're ready to submit your revision, log on to https://www.editorialmanager.com/pdig/ and select the 'Submissions Needing Revision' folder to locate your manuscript file. Please include the following items when submitting your revised manuscript:* A rebuttal letter that responds to each point raised by the editor and reviewer(s). You should upload this letter as a separate file labeled 'Response to Reviewers '. This file does not need to include responses to any formatting updates and technical items listed in the 'Journal Requirements' section below.* A marked-up copy of your manuscript that highlights changes made to the original version. You should upload this as a separate file labeled 'Revised Manuscript with Track Changes '.* An unmarked version of your revised paper without tracked changes. You should upload this as a separate file labeled 'Manuscript '. If you would like to make changes to your financial disclosure, competing interests statement, or data availability statement, please make these updates within the submission form at the time of resubmission. Guidelines for resubmitting your figure files are available below the reviewer comments at the end of this letter. We look forward to receiving your revised manuscript. Kind regards, Francesco Andrea CausioGuest EditorPLOS Digital Health Francesco Andrea CausioGuest EditorPLOS Digital Health Leo Anthony CeliEditor-in-ChiefPLOS Digital Healthorcid.org/0000-0001-6712-6626  **Additional Editor Comments (if provided):** Dear Authors,

Thank you for submitting your manuscript to PLOS Digital Health. After carefully reviewing your work, I find it to be moderately interesting and a valuable contribution to the field. I believe it is suitable for publication in the journal, provided that a few minor revisions are addressed.

Please take the time to carefully address the comments and suggestions provided in the review. Once these changes have been implemented, I am confident that the manuscript will meet the standards required for publication. [Note: HTML markup is below. Please do not edit.] **Reviewers' Comments:** Reviewer's Responses to Questions

**Comments to the Author**

1. Does this manuscript meet PLOS Digital Health’s publication criteria ? Is the manuscript technically sound, and do the data support the conclusions? The manuscript must describe methodologically and ethically rigorous research with conclusions that are appropriately drawn based on the data presented.

Reviewer #1: Yes

Reviewer #2: Yes

**********

2. Has the statistical analysis been performed appropriately and rigorously?

Reviewer #1: N/A

Reviewer #2: I don't know

**********

3. Have the authors made all data underlying the findings in their manuscript fully available (please refer to the Data Availability Statement at the start of the manuscript PDF file)?

The PLOS Data policy requires authors to make all data underlying the findings described in their manuscript fully available without restriction, with rare exception. The data should be provided as part of the manuscript or its supporting information, or deposited to a public repository. For example, in addition to summary statistics, the data points behind means, medians and variance measures should be available. If there are restrictions on publicly sharing data—e.g. participant privacy or use of data from a third party—those must be specified.

Reviewer #1: Yes

Reviewer #2: Yes

**********

4. Is the manuscript presented in an intelligible fashion and written in standard English?

PLOS Digital Health does not copyedit accepted manuscripts, so the language in submitted articles must be clear, correct, and unambiguous. Any typographical or grammatical errors should be corrected at revision, so please note any specific errors here.

Reviewer #1: Yes

Reviewer #2: Yes

**********

5. Review Comments to the Author

Please use the space provided to explain your answers to the questions above. You may also include additional comments for the author, including concerns about dual publication, research ethics, or publication ethics. (Please upload your review as an attachment if it exceeds 20,000 characters)

Reviewer #1: In my opinion the paper fits with PLOS requirements, I thin that the requirement "statistical analysis been performed appropriately and rigorously" isn't applicable because it is a qualitative reasearch and there aren't enough references to assess this point. In the introduction there is a light disclosure about the composition of the cluster of patients that seem to be representative of adults population but I don't think that the parameter of statistical analysis can be applied to this paper

Reviewer #2: General:

Language revision is required. Sentences should be concise and to the point, authors tend to use long sentences which are -sometimes- confusing.

The abbreviation HCS is written as HSC many times. Is this a typo or does it refer to another term? Please explain.

Title:

Title of the study is long and does not capture the study method and purpose. Please modify.

Abstract:

Avoid using abbreviations in the abstract section.

Introduction:

Page 8 line 96: I am not sure what the brackets here refer to.

Themes:

I would not use the word "challenges" for every dissatisfying quality of WT. Authors should reconsider that label.

**********

6. PLOS authors have the option to publish the peer review history of their article (what does this mean? ). If published, this will include your full peer review and any attached files.

**Do you want your identity to be public for this peer review?** If you choose “no”, your identity will remain anonymous but your review may still be made public.

For information about this choice, including consent withdrawal, please see our Privacy Policy .

Reviewer #1: **Yes: ** giulia cartei

Reviewer #2: **Yes: ** Samia A. Abdul-Rahman

**********

 [NOTE: If reviewer comments were submitted as an attachment file, they will be attached to this email and accessible via the submission site. Please log into your account, locate the manuscript record, and check for the action link "View Attachments". If this link does not appear, there are no attachment files.] **Figure resubmission:** While revising your submission, please upload your figure files to the Preflight Analysis and Conversion Engine (PACE) digital diagnostic tool, https://pacev2.apexcovantage.com/. PACE helps ensure that figures meet PLOS requirements. To use PACE, you must first register as a user. Registration is free. Then, login and navigate to the UPLOAD tab, where you will find detailed instructions on how to use the tool. If you encounter any issues or have any questions when using PACE, please email PLOS at figures@plos.org. Please note that Supporting Information files do not need this step. If there are other versions of figure files still present in your submission file inventory at resubmission, please replace them with the PACE-processed versions. **Reproducibility:** To enhance the reproducibility of your results, we recommend that authors of applicable studies deposit laboratory protocols in protocols.io, where a protocol can be assigned its own identifier (DOI) such that it can be cited independently in the future. Additionally, PLOS ONE offers an option to publish peer-reviewed clinical study protocols. Read more information on sharing protocols at https://plos.org/protocols?utm_medium=editorial-email&utm_source=authorletters&utm_campaign=protocols

Attachment Submitted filename: Plos Digital Health_review paper.pdf

# Author comment: Author response to Decision Letter 0

**DOI:** 10.1371/journal.pdig.0000844.r003

24 Mar 2025

Attachment Submitted filename: Rebuttal letter Svardh et al WT interview study.docx

# Editor report: Decision Letter 1

**DOI:** 10.1371/journal.pdig.0000844.r004

**Authors:** Francesco Andrea Causio

1 Apr 2025

"It is very convenient when it works - sucesses and challenges with welfare technology - a qualitative study"

PDIG-D-25-00045R1

Dear Mrs. Svärdh,

We are pleased to inform you that your manuscript '"It is very convenient when it works - sucesses and challenges with welfare technology - a qualitative study"' has been provisionally accepted for publication in PLOS Digital Health.

Before your manuscript can be formally accepted you will need to complete some formatting changes, which you will receive in a follow-up email from a member of our team. 

Please note that your manuscript will not be scheduled for publication until you have made the required changes, so a swift response is appreciated.

IMPORTANT: The editorial review process is now complete. PLOS will only permit corrections to spelling, formatting or significant scientific errors from this point onwards. Requests for major changes, or any which affect the scientific understanding of your work, will cause delays to the publication date of your manuscript.

If your institution or institutions have a press office, please notify them about your upcoming paper to help maximize its impact. If they'll be preparing press materials, please inform our press team as soon as possible -- no later than 48 hours after receiving the formal acceptance. Your manuscript will remain under strict press embargo until 2 pm Eastern Time on the date of publication. For more information, please contact digitalhealth@plos.org.

Thank you again for supporting Open Access publishing; we are looking forward to publishing your work in PLOS Digital Health.

Best regards,

Francesco Andrea Causio

Guest Editor

PLOS Digital Health

***********************************************************

**Additional Editor Comments (if provided):**

**Reviewer Comments (if any, and for reference):**
